# Seasonal Influenza Vaccination Uptake Among Australian Healthcare Professionals: An Archetype for Success

**DOI:** 10.3390/vaccines13010071

**Published:** 2025-01-14

**Authors:** Caroline M. Hall, Anthony Cotton, Adrian Webster, Mary Bushell, Holly L. Northam

**Affiliations:** 1 Faculty of Health, University of Canberra, Bruce, ACT 2617, Australia; adrian.webster@aihw.gov.au (A.W.); mary.bushell@canberra.edu.au (M.B.); holly.northam@canberra.edu.au (H.L.N.); 2 School of Management, UNSW Canberra, Canberra, ACT 2612, Australia; z5023663@ad.unsw.edu.au; 3 Australian Institute of Health and Welfare, Bruce, ACT 2617, Australia

**Keywords:** healthcare professional, vaccination, seasonal influenza, archetype, behaviour change

## Abstract

Background/Objectives: Qualitative research suggests there may be identifiable characteristics that form a health professional (HCP) archetype associated with habitual seasonal influenza vaccination (SIV). However, the validity of this archetype requires further investigation, ideally within a theoretical framework that can elucidate this association and its generalisability to other vaccines. This study aims to confirm key HCP archetype characteristics associated with SIV, as informed by prior qualitative research findings, and test the generalisability of the association between this archetype and SIV to COVID-19 vaccine acceptance. Method: A cross-sectional survey was designed and distributed to an Australian HCP sample consisting of practicing nurses, midwives, pharmacists, and medical practitioners. The anonymous online survey measured key characteristics that predict vaccination behaviour and intention. Results: Most participants (n = 173) demonstrated habitual SIV behaviour (77.91%) associated with the intention to vaccinate in the future. Survey findings supported the HCP archetype, as key constructs were associated with vaccination intention and behaviour, including heightened professional responsibility, vaccine confidence, and protection of self and patients. Furthermore, results suggested progressing vaccination intention to behaviour, overcoming vaccine complacency, is possible through the provision of free, accessible vaccination services. These critical factors were broadly generalisable to the COVID-19 vaccine. Conclusions: A vaccination-positive HCP archetype, supported by access to free, convenient vaccination services, was associated with the likelihood of future vaccination behaviour, including in future pandemic response scenarios. However, it will be important to ensure that HCP vaccine knowledge gaps are minimised to enhance trust in this cohort to enable broad success.

## 1. Introduction

In Australia, healthcare professionals (HCPs) are encouraged, and in clinical settings mandated, to receive seasonal influenza vaccination (SIV) as a key measure to manage occupational risk, safeguard productivity and enhance patient safety [1,2]. HCPs are at increased risk of exposure to influenza and other circulating viruses through their interactions with patients and the healthcare environment. SIV provides a degree of protection from ‘the flu,’ reducing the risk of nosocomial and community transmission and galvanising its importance in infection control and occupational health and safety protocols [1].

Recent qualitative research [3] suggests the existence of a distinct health professional archetype that is associated with SIV uptake, characterised by specific identifiable attitudes, beliefs, professional and personal knowledge, and experiences. This archetype appears to support very high levels of SIV uptake when provided with easily accessible and convenient vaccination services [3]. Positioned within the context of a theoretical framework—Triandis’ theory of interpersonal behaviour (TIB) [4]—these findings suggest the archetype may offer valuable insights into key constructs that influence vaccine decision-making. Specifically, the influence of this archetype might be:

- to isolate significant constructs that inform interventions to encourage vaccine uptake,
- to generalise this concept to vaccines for other emerging infectious diseases (EIDs).

In the absence of recent Australian studies exploring factors that influence SIV behaviour among this highly exposed professional cohort, the potential existence of such an archetype and its relationship with vaccine uptake requires further confirmation. This can be achieved through the analysis of a larger, more diverse sample.

The Australian Technical Advisory Group on Immunisation (ATAGI) recommends annual influenza vaccination for all healthcare workers and carers who may transmit the virus to high-risk groups vulnerable to serious complications [5]. Similarly, in response to the COVID-19 pandemic, all Australians aged 18 years and over are recommended the primary vaccination course for SARS-CoV-2 [6]. Given the evolving nature of national vaccination policies and mandates, with variation across settings and jurisdictions [7,8], understanding factors that shape HCP-positive vaccination behaviour is crucial to mitigate vaccine hesitancy and complacency.

Building on semi-structured interviews of HCPs, Triandis’ TIB framework was applied to explore SIV decision-making processes [3]. Through reflexive thematic analysis [9], this study identified specific constructs, a typical ‘archetype’, which included:

• professional responsibility—strong identification of social norms aligning to professional roles that are positive toward vaccine uptake,
• protection—strong attitudes towards patient protection, followed by protection of self, including family,
• vaccine confidence—confidence in vaccine safety and reasonable effectiveness, underpinned by trust in vaccine governance and regulatory bodies (e.g., Therapeutic Goods Administration),
• knowledge and experience—of the disease, its sequalae and the vaccine, and
• habit—high frequency of past vaccination behaviours,
• Supporting the archetype characteristics, vaccine convenience was also identified as a key component of vaccine uptake, overcoming complacency and translating vaccination intention to behaviour [3].

The aim of this study was to address a contemporaneous gap in health decision-making knowledge from the Australian context by examining HCP’s vaccine uptake using this unique approach. We aim to validate earlier qualitative findings and test the generalisability of the archetype in the context of emerging diseases, novel vaccines, and new technologies. Study findings will aid in the identification of upstream policy initiatives that support both seasonal and pandemic vaccination efforts through understanding HCP alignment with the identified archetype.

## 2. Materials and Methods

### 2.1. Study Design

A cross-sectional survey design was implemented, using the prior qualitative research to inform survey development [3], with questions designed to test the constructs found to be associated with HCP SIV uptake. Demographic questions were adapted from the ‘Behavioural and social drivers of vaccination (BeSD-COVID-19) survey for adults and health workers (1.0) [10]’, a globally standardised tool, which was modified for the online survey platform.

Following human research ethics committee approval from the University of Canberra Human Research Ethics Committee, the anonymous online survey was piloted in November 2023 with 12 known participants to assess language clarity, survey development and delivery, and content validity. Feedback from this pilot phase streamlined the survey design and clarified any ambiguous response options (Supplementary Materials).

The final online survey consisted of three sections.

(1) Demographic Information: This section gathered data on participants’ location, age, gender, profession, identification with First Nation people’s heritage, years of professional experience, and type of employment. It also categorised participants’ direct patient or clinical interactions.
(2) Influenza Vaccination: The section explored participants’ attitudes, beliefs, vaccination history, future intentions, and barriers to accessing vaccination services. The domains of experience and knowledge, trust and vaccine confidence, risk perception and concern, professional responsibility and role, and protection were tested, with follow-up questions identifying vaccine access, modes of communication, and service delivery.
(3) COVID-19 vaccination: Participants were asked about their history of COVID-19 vaccine uptake, future intentions, enablers, and barriers to vaccination.

Participants were asked to identify reasons for vaccination and non-vaccination in pre-determined responses and free text. Confidentiality of participant responses was assured throughout all stages of participant recruitment, data collection and analysis.

### 2.2. Measures

The survey measured key constructs critical to HCPs vaccination decision-making through a series of items:

• Vaccination behaviour and intention (two items),
• Reason for receiving vaccination, non-vaccination (top three reasons),
• Convenience and ease of access to vaccination services (seven items, including communications and knowledge of distribution methods),
• Affect: trust, risk perception, and fear (eight items),
• Social Norms: professional responsibility, role, and social identity (eight items),
• Knowledge and experience (six items),
• Vaccine confidence and protection (eight items),

### 2.3. Study Population and Procedure

As an exploratory investigation, with no fixed hypotheses and no a priori estimates of effect size for the comparisons to be made, there was no fixed minimum sample size determined for the study. However, an estimate of an effective sample size for the analysis of 57 participants was obtained using the R statistical software package (version 4.4.2, pwr.chisq.test function from the pwr library) using the following parameters:

• Power = 0.8
• Alpha = 0.5
• Effect = 0.3 (moderate)
• Degrees of Freedom = 12—the most complex Chi-square analysis would compare three groups (vaccine-accepting, vaccine-complacent/hesitant, and vaccine-refusing) across survey questions with seven potential responses, yielding twelve degrees of freedom.

A total of 173 Australian HCP participants consented to this anonymous, voluntary online survey, open from December 2023 to April 2024. The study sample, which was broadly representative of HCP populations, included enrolled/registered nurses and dual registration (midwifery and nursing), registered midwives, medical practitioners, and pharmacists. Participants with less than 60% survey completion were excluded. All participants recruited were over 18 years of age from across Australia, with representation from all states and territories in Australia.

### 2.4. Data Collection

Survey promotion involved distributing posters, adverts, and flyers with a descriptive ‘brief’ and an embedded QR code and survey link distributed through professional organisations, social media platforms, and direct emails to healthcare facilities and businesses. Participant information outlining study objectives and expected research outputs preceded the consent form and survey access via Qualtrics. Access to the survey was only granted once informed consent was obtained. Participation was voluntary and anonymous.

After completing the SIV section (section two), participants could choose to continue with COVID-19 questions (section three). If participants indicated positive vaccination behaviour for sections two and three, they were prompted to provide their ‘reasons for vaccination’. ‘Reasons for non-vaccination’ followed a negative response to vaccination behaviour.

### 2.5. Statistical Analysis

After data cleaning, descriptive and inferential statistical analyses were performed using Stata IC version 16.1. In the reporting of Likert-type items, only the ‘strongly agree’ or ‘agree’ (and conversely ‘strongly disagree’ and ‘disagree’) categories are used to reflect agreement (or disagreement) with a statement. Responses indicating participants only ‘somewhat agreed’ (or ‘somewhat disagreed’) are not reported as they are considered to reflect response uncertainty rather than a firm attitude.

## 3. Results

### 3.1. Participant Characteristics

In total, 173 consented responses were analysed. Females comprised the majority (n = 134, 77.7%), with males (n = 34, 20.0%), non-binary individuals (n = 1, 0.6%), and those preferring not to disclose (n = 3, 1.8%). Nurses were the most common health professionals in the sample (57.49%), followed by medical practitioners (23.35%), pharmacists (11.38%), and midwives (7.78%). Most participants were working full-time (58.82%), most had direct patient contact in their role (71.60%), and most worked in either a hospital (47.93%) or primary or community health care (24.85%).

### 3.2. Vaccination Behaviour and Intention

Vaccination behaviour was measured by asking participants about their SIV uptake for the current year. Additionally, participants were asked about their usual SIV behaviour, categorising vaccine uptake responses into most years, some years, or having never had SIV (Table 1).

Responses were then collapsed into three categories to indicate habitual vaccination behaviour:

- those who were vaccine-accepting (had the vaccine most years),
- those who were complacent or hesitant (had the vaccine some years), and
- those who were vaccine-refusing (had never had the vaccine).

Vaccination-accepting HCPs, those receiving the vaccines most or every year, consisted of 77.91% of the sample. While complacent or hesitant HCPs (16.56%) had lower levels of SIV uptake, and 5.52% of HCPs refused vaccination completely.

Habitual vaccination behaviour was then compared across several key characteristics of the participants. This showed that there were no statistically significant associations between habitual vaccination behaviour and the type of healthcare professional—nurses, midwives, medical practitioners, and pharmacists (X^2^ = 6.28, df = 6, *p* = 0.39); participant gender (X^2^ = 3.29, df = 6, *p* = 0.77); whether the participant had seasonal influenza previously (X^2^ = 26.53, df = 6, *p* = 0.00); or in which healthcare domain they worked (X^2^ = 7.79, df = 8, *p* = 0.45). However, it did show a statistically significant association between habitual vaccination behaviour and expressed intention to have SIV next year (X^2^ = 88.83, df = 4, *p* = 0.00). Most participants (82.55%) intended to have SIV next year (n = 123).

### 3.3. Reasons for Receiving SIV, and Non-Vaccination

Participants provided their top three reasons for obtaining SIV. The most often cited reasons were as follows (Table 2):

### 3.4. Convenience and Access

Participants generally found SIV accessible and convenient. Participants felt that time was made available to obtain SIV (61.88% agreed), they understood where to access vaccination services (97.50%), and for most, it was available at their place of work (88.12%) and was cost-free (88.68%). Participants were asked to identify barriers to accessing vaccination services, with more than half (66.46%) stating they experienced no difficulties. Participants were informed of SIV availability primarily through email reminders from their organisation (124 responses), posters or brochures in the workplace (65 responses), and their manager or supervisor (55 responses). However, habitual vaccination was only significantly associated with the availability of the SIV in the workplace (X^2^ = 16.93, df = 6, *p* = 0.01) and whether it was available for free (X^2^ = 19.82, df = 4, *p* = 0.00).

### 3.5. Risk Perception and Trust

Participants were asked a range of questions relating to their perceptions of risk associated with SIV. Most participants expressed heightened risk perception related to contracting seasonal influenza, with 76.83% expressing some degree of concern about becoming ill.

Trust in disease information, vaccine development, and vaccine regulatory frameworks may influence risk perception and vaccine confidence. The survey probed HCPs’ trust in these areas. Australian HCPs conveyed trust in the Therapeutic Goods Administration (TGA: 73.41%) and the provision of reliable information about vaccine risks and benefits from authorities (73.13%). They predominantly disagreed with statements proposing that SIV is promoted for financial gain (73.88%), that natural exposure to disease is safer for the immune system than being exposed through vaccination (60.84%), and they disagreed with the notion that HCPs were less susceptible to the flu than others (72.79%). Moreover, habitual vaccination behaviour was related to participants’ views on each of the factors related to risk perception and trust (Table 3).

### 3.6. Vaccination as a Social Norm: Professional Responsibility and Role

Two of the four most cited reasons given by participants for obtaining the SIV were related to professional responsibility: As a healthcare professional, it is important that I get the flu vaccine, and it is part of my professional role and professional responsibility to have the seasonal flu vaccine. Moreover, vaccine compliance was significantly associated with preparedness to recommend SIV to others, another marker of professional responsibility (X^2^ = 74.14, df = 12, *p* = 0.00).

### 3.7. Vaccine Confidence and Protection

Confidence in influenza vaccines was high, with most agreeing or strongly agreeing that the vaccines were safe (85.27%) and effective in minimising influenza (70.19%). This reflects strong overall vaccine confidence, though some participants retained caution about potential long-term safety (22.73%). Moreover, all of these attitudes, except for the belief that the vaccine will not give the participant influenza, were significantly associated with habitual vaccination behaviour (Table 4).

### 3.8. Vaccination Knowledge and Experience

Participants demonstrated an understanding and knowledge of the flu vaccine. Participants agreed that obtaining the SIV does not give them the flu (85.1%), they understood how the vaccine protects them from influenza (78.39%), and they could answer patients’ questions about SIV (73.62%). None of these attitudes was associated with habitual vaccination behaviour (Table 5).

### 3.9. COVID-19 Vaccine Findings

A total of 145 participants completed the questions related to COVID-19 vaccination behaviour; of these, 111 (76.55%) indicated they had previously contracted COVID-19, and most felt some degree of concern about further COVID-19 infections (82.07%).

Among all participants, 94.5% had acquired the full primary course of COVID-19 vaccines (Table 6). While 14.5% of respondents stated mandates were instrumental in vaccination compliance, 11% stated they were unaware of current COVID-19 vaccine recommendations for HCP (vaccination requirements).

Participants listed their top three reasons, beyond the public health mandates, for obtaining COVID-19 vaccines; most cited reasons are shown in Table 7.

Top reasons for vaccinating prioritised protection and professional role and responsibility. Participants intention to vaccinate against COVID-19 in the future saw 53.15% of participants affirm future vaccination intention (Table 8).

## 4. Discussion

### 4.1. Vaccination-Supporting Archetype

Archetypes can serve as valuable tools for identifying key factors that influence health decision-making, offering insights into how tailored interventions can support unique priorities and contexts [11]. We believe applying this unique approach will, as a positive-vaccinating archetype, help isolate key constructs influencing SIV behaviour. Our earlier qualitative research highlighted HCP characteristics—such as values, beliefs, and knowledge—that are associated with higher SIV uptake [3]. The survey results presented here support these findings and reveal the presence of a distinct HCP archetype that is associated with SIV uptake. Beyond the archetype characteristics, achieving improved SIV uptake in HCPs seems associated with an organisation’s or employer’s ability to provide convenient and highly visible vaccination services free of charge.

### 4.2. Professional Responsibility- Identity and Social Norms

For Australian HCPs, the results highlighted their professional responsibilities to inform their vaccination behaviour. The prior qualitative research predicted that HCPs identifying vaccination as a social norm and professional responsibility would demonstrate habitual vaccine acceptance. Survey data analysis supports an association between professional responsibility and vaccination uptake. Similar results were identified in European, North American, and Southeast Asian studies where group professional identification and duty were significant predictors of health-protective behaviours [12,13,14,15].

The Australian government and health authorities recognise healthcare workers as a high-risk cohort due to their occupational exposure [5]. The archetype HCP appreciates and voices concern about the occupational risk associated with the professional role, acknowledging the need for protection. Leveraging this insight, emphasizing HCP professionalism, the related behavioural norms, and their importance, especially in early career development, education, and ongoing training, may improve SIV uptake. Fostering HCP professionalism while enhancing trust may benefit organisations by stressing the importance of patient- and self-protection, as well as promoting habit-forming self-protective practices.

### 4.3. Trust, Vaccine Confidence, and Protection

The concept of trust and vaccine confidence has long been interwoven into the vaccine hesitancy discourse. Vaccine confidence encompasses trust in vaccine safety and effectiveness, delivery systems, the reliability and competence of health services, health professionals, and public health policy [16,17]. HCPs demonstrated belief in SIV safety and effectiveness in the vaccine’s ability to protect themselves and their patients. Participants identified protection of self and patients as a motivator for SIV. Regardless of protection focus—self, patient, or family—confidence in the protection afforded by SIV appears instrumental in vaccination intention, as identified in other literature [18,19,20,21]. This is despite HCPs’ knowledge and understanding of SIV variable effectiveness, which is often comparatively lower than other vaccines [22].

Trust in vaccine governance was reported as associated with vaccination behaviour in just under three-quarters of participants. Trust in institutions that combat emerging diseases is important for vaccination uptake [23]. These findings reflect similar attitudes in many Asian countries where a willingness to receive the COVID-19 vaccines was associated with strong trust in central governments [24]. For Australian HCPs, improved communications highlighting the role of institutions of vaccine governance and regulatory bodies, such as the Therapeutic Goods Administration (TGA), may nudge and further encourage those with uncertainty due to incomplete knowledge (approximately 26% of HCPs) to trust, bolstering vaccine acceptance and potentially reducing vaccine scepticism [23]. While the number of non-vaccinating HCPs in this study was small, they expressed significant distrust in vaccines and institutions responsible for vaccine governance, official information, and multi-national players such as pharmaceutical companies. This aligns with findings from other studies, where some HCPs expressed scepticism and mistrust in governance institutions and pharmaceutical companies due to perceived violations of trust relating to the influence of financial incentives or profits over safety, historical injustices, or malpractice reporting, which has fuelled vaccine hesitancy or vaccine refusal [25,26,27,28,29,30]. This portrays a larger socio-political dynamic beyond vaccination choices.

### 4.4. Generalisability of the Archetype

Survey results suggest the HCP archetype characteristics associated with professional responsibility, vaccine confidence, risk perception, and ease of access may support the uptake of vaccines administered in other contexts, including emergency response situations similar to the COVID-19 pandemic. Australian HCP COVID-19 vaccination uptake of the required full primary COVID-19 vaccine course (94.5%) aligned with community vaccination compliance [31]. However, our results found future intention for COVID-19 booster vaccination (53%) is significantly lower than SIV future intention (82%). Further research is required to understand the unique Australian HCP hesitance around future COVID-19 vaccines. Although a range of influences may impact future COVID-19 vaccination intention, participants expressed uncertainty around vaccination booster schedules (reduced knowledge) and organisational expectations (professional responsibility) for COVID-19 HCP vaccine coverage.

In the absence of a global pandemic to drive demand, further COVID-19 vaccines and other novel vaccine technologies may require robust, multi-faceted communication strategies and ongoing education to improve vaccine confidence. Strong advocacy from HCPs to foster community vaccine confidence and support the intention to vaccinate in the future will require education and knowledge sharing around novel vaccine technologies, for example, mRNA vaccines, to enhance trust. As suggested by archetypical characteristics, low vaccine confidence, with suboptimal knowledge and experience with novel vaccines, may impede future vaccination intention and potential vaccination promotion, especially in the context of emerging technologies, which some HCPs are hesitant to trust [8,29]. Therefore, it will be important to ensure that HCP knowledge gaps are minimised if broad success in vaccination is to be achieved.

In summary, the vaccinating-positive HCP archetype displays habitual uptake and a strong intention to continue vaccination in the future. This is facilitated by trust in the vaccine, its development, and its governance, which is associated with vaccine confidence. HCPs who demonstrated this archetype are aware of their professional responsibility and the importance of vaccination to protect themselves and others. They express concerns about disease exposure, understand conventional vaccines, and have solid knowledge or experience of the disease. The archetype’s vaccination behaviour is enabled by on-site, workplace vaccination services that are cost-free. Without these identified characteristics or enabling constructs, the likelihood of vaccination intention and behaviour is diminished. It is reasonable to predict that both the archetype characteristics and convenience factors associated with SIV uptake may generalise to support HCP uptake of other vaccines.

### 4.5. Recommendations

The archetype concept suggests ongoing leveraging of specific constructs to facilitate positive vaccination behaviours. For example, HCP professionalism could be emphasised through professional education and training programs to highlight the importance of their role as models, advocates for, and champions of community uptake of SIV. The conclusions from this study are consistent with other evidence that suggests upstream measures for promoting strong nursing professionalism, for example, and trust in health governance and regulatory institutions to support vaccination behaviour [23]. These findings also suggest that high SIV uptake may be supported by ensuring vaccination service access is convenient and free of charge.

This research provides evidence that supports the integration of core professional values, responsibilities, and expectations around vaccinations into HCP education curricula, as well as early career development and training and ongoing professional development. We recommend further studies to evaluate the impact of embedding vaccination education within student curricula targeted at the archetype. Noting vaccination is often a requirement for practical placements, we recommend organisational practices that help establish trust through evidence and knowledge sharing while reinforcing the importance of both patient and self-protection. As vaccine technologies evolve, ongoing contemporaneous education for HCPs, particularly regarding mRNA vaccines and vaccine regulatory processes, will be crucial for enhancing trust and confidence, and therefore, HCP acceptance in future COVID-19 vaccines and other novel vaccines, such as those designed to combat seasonal influenza.

From a pandemic preparedness perspective, routinely monitoring the alignment of HCPs with established archetype constructs could serve as a valuable predictor of HCP vaccine acceptance and uptake in the context of disease outbreaks. For example, if there is a noted shift over time at a population level of growing HCP misalignment from key constructs or characteristics of the archetype, this may signal a reduced likelihood of vaccine uptake by HCPs in the event of a future disease outbreak.

### 4.6. Limitations of This Study

This study has some limitations. Firstly, although a diverse cohort responded to the survey, the sample size is small, potentially limiting the generalisability of the findings. There is also the risk of volunteer bias, with more interested participants completing the survey and non-vaccinating HCPs not wishing to engage, limiting conclusions about reasons for non-vaccination. To counter this, diverse recruitment strategies were employed, including online, email, and social media recruitment, with various healthcare personnel and facilities approached to mitigate biases.

Surveys examining vaccination behaviour have also been plentiful in the post-COVID-19 era, contributing to survey fatigue. Furthermore, the slightly off-set timing for this survey with the Australian SIV season may be responsible for recall bias, especially around vaccination uptake when the pandemic has interrupted normal vaccination routines. These biases were addressed by asking HCPs about their ‘usual’ vaccination behaviour over a number of years and ensuring the survey design was as ‘participant-friendly’ as possible. This was achieved through the pilot study that ensured the survey design minimised participant burden, unclear questions, or technical issues in the survey instrument.

Australian regulatory bodies may proceed with regulatory action [32] if anti-vaccination rhetoric is attributed to HCPs. In studies examining vaccination attitudes and intentions, this may stifle authentic and robust discussions. It may also minimise vaccination survey participation if there is a fear of non-anonymity.

Furthermore, this study only establishes correlation, not causation. The archetype states there are associations between belief or attitude and actions, but the direction is undetermined. Despite the limitations, the findings are valuable and add to the SIV and COVID-19 discourse from the unique Australian context.

## 5. Conclusions

For Australian HCPs, vaccination decision-making is not based purely on knowledge about the disease or the vaccine. Most HCPs expressed confidence in the vaccine and trust in vaccination operational frameworks and governance. The survey data also associate vaccine accessibility and confidence with successful SIV uptake. The application of a vaccination-positive HCP archetype may aid in the identification of key characteristics that, when leveraged, improve vaccine uptake by HCPs.

These findings, suggesting a positive-vaccinating archetype, were extrapolated and compared in efforts to better understand HCP SIV and COVID-19 vaccination acceptance, particularly as public health orders have eased and ambiguity around vaccination protocols impacts health decision-making. Addressing vaccine complacency through a focus on positive archetype characteristics may promote SIV, while greater emphasis must be placed on addressing vaccine hesitancy within the COVID-19 vaccine discourse, as HCP intention for future vaccine uptake remains uncertain. This is crucial in supporting vaccination intention and behaviour in both HCP and community populations in the context of emerging infectious diseases on our horizon, which will require a protected workforce reliant on old and new vaccine technologies.

## Figures and Tables

**Table 1:** Vaccination Behaviour.

Vaccination Behaviour	Response (%)
Received the vaccine this year and most years	49.08%
Received the vaccine this year and some years	6.13%
Did not receive the vaccine this year but most years	28.83%
Did not receive the vaccine this year but some years	10.43%
No vaccination—not this year, not ever	5.52%

**Table 2:** Vaccination reasoning.

Reason for Seasonal Influenza Vaccination	Frequency (n)
Belief in personal health protection provided by the flu vaccine.	109
As a healthcare professional, it is important that I get the flu vaccine.	79
It is part of my professional role and a professional responsibility to have the seasonal flu vaccine.	53
Belief that I can infect my patients with the flu if I do not get the flu vaccine.	47

**Table 3:** Habitual vaccination behaviour related to risk perception and trust.

Risk Perception and Trust Factor	Response
Authorities promote SIV for financial gain	*X*^2^ = 60.18, *df* = 10, *p* = 0.00
Natural exposure is safer	*X*^2^ = 50.94, *df* = 12, *p* = 0.00
Trust in the TGA	*X*^2^ = 55.55, *df* = 12, *p* = 0.00
Trust in receiving reliable information	*X*^2^ = 65.12, *df* = 12, *p* = 0.00
Healthcare professionals are less susceptible	*X*^2^ = 27.07, *df* = 10, *p* = 0.00
Concern about contracting the flu	*X*^2^ = 26.54, *df* = 6, *p* = 0.00

**Table 4:** Habitual vaccination behaviour related to vaccine confidence and protection.

Vaccine Confidence and Protection	Response
Vaccine is safe	*X*^2^ = 78.98, *df* = 12, *p* = 0.00
Vaccine is effective	*X*^2^ = 60.38, *df* = 12, *p* = 0.00
Possible future problems with vaccines	*X*^2^ = 49.05, *df* = 12, *p* = 0.00

**Table 5:** Habitual vaccination behaviour related to vaccine knowledge and experience.

Vaccine Knowledge and Experience	Response
Vaccine will not cause influenza	*X*^2^ = 19.33, *df* = 12, *p* = 0.08
Understand how vaccine protects	*X*^2^ = 15.09, *df* = 12, *p* = 0.13
Can answer patient’s questions	*X*^2^ = 15.37, *df* = 12, *p* = 0.22

**Table 6:** COVID-19 Vaccination Behaviour.

Vaccination Behaviour	Responses (%)
Yes, I will have had all the vaccines (Primary course + Boosters)	48.97%
Yes, I have had a number of the vaccines (Primary course ± Boosters)	45.52%
Yes, I have had one of the vaccines	0.69%
No, not ever	4.83%

**Table 7:** COVID-19 most cited reasons for vaccination (max 3).

COVID-19 Vaccination Behaviour	Frequency (n)
I personally believe that having the COVID-19 vaccines will protect my health	85
As a healthcare professional, it is important that I get the COVID-19 vaccines	61
If I get vaccinated against COVID-19, then I will be more certain that I will not infect my family	55
It is part of my professional role and a professional responsibility to have the COVID-19 vaccines	53
It is very likely that I can infect my patients with COVID-19 if I do not get the vaccines	33

**Table 8:** COVID-19 intention to vaccinate.

Intention to Vaccinate	Response (%)
Yes	53.15%
No	18.88%
Maybe	27.97%

## Data Availability

Data supporting reported results can be found at DOI:10.17632/ddvgdn7v7w.1.

## Supplementary Materials

The following supporting information can be downloaded at: https://www.mdpi.com/article/10.3390/vaccines13010071/s1. Australian healthcare professionals and seasonal influenza vaccination survey.
