# Supplementary material for: Seasonal Influenza Vaccination Uptake Among Australian Healthcare Professionals: An Archetype for Success
Source: Vaccines (Basel). 2025 Jan 14;13(1):71. doi: 10.3390/vaccines13010071 (PMC11769453; doi:10.3390/vaccines13010071)
Supplement: Supplementary file 1 [file vaccines-13-00071-s001.zip › vaccines-3391620-supplementary.pdf]

Appendix 1: Australian healthcare professionals and seasonal influenza vaccination survey

SECTION 1: Demographics

1.1 How would you describe your Employment location? Please choose which category/ area your main job falls under.

Area	Description
**Metropolitan areas**	All Major cities.
**Regional Centre**	Inner and Outer Regional areas that are in, or within a 20km drive of a large centre/town with over 50,000 residents.<br>For example: Ballarat, Mackay, Toowoomba, Kiama, Albury, Bunbury.
**Rural Area (Large-small towns)**	Inner and Outer Regional areas that are within a 15km drive of a small, medium, or large rural town (under 50,000 residents).<br>For example: Dubbo, Lismore, Yeppoon, Busselton, Port Augusta, Charters Towers, Moree, Mount Buller, Moruya, Renmark, Condamine.
**Remote/ Very Remote communities**	Remote mainland areas and islands.<br>For example: Cape Tribulation, Lightning Ridge, Alice Springs, Mallacoota, Port Hedland, Longreach, Coober Pedy, Thursday Island and all other remote island areas
**No usual address**	Variable / mobile work arrangement.

1.2 What State/ Territory do you work in? ______________________

1.3 What is your age? _________________

1.4 To which Gender Identity do you most identify? Please highlight.

**Man**	**Woman**
**Non-Binary**	**I'd prefer not to say**
**Other**	

1.5 Do you identify as Aboriginal or Torres Strait Islander? ____________________

1.6 Which Professional Category applies to you? Please highlight all those applicable.

**Registered Nurse**	**Registered Midwife**
**Student Nurse**	**Student Midwife**
**Enrolled Nurse**	**Pharmacist**
**Medical Officer**	**Pharmacy student**
**Medical Student**	**Dual (above + other not listed)**

1.7 Which best describes your employment status?

**Employed, full-time**	**Employed, part-time.**
**Employed, casual**	**Not employed, looking for work.**
**Student, full-time**	**Student, part-time**
**Not employed, not looking for work.**	**Other______________________**

1.8 Number of years in your current profession? _________________

1.9 Do you have patient/ client contact or clinical interactions? ANS: Yes, No, yes, but infrequently,

1.10 Healthcare domain: Please choose which apply/applies to you. (AIHW)

**Health Promotion and Health Protection**	e.g., Immunisation services, health initiatives and programs, cancer screening
**Primary Health Care**	e.g., General Practice, Community Pharmacies, allied health practices, Community Health Centres, walk-in centres.
**Specialist Care**	e.g., Specialist care, palliative care, drug and alcohol services, imaging and pathology services,
**Hospitals**	e.g., All admitted and non-admitted patient services including outpatients and Emergency Department.
**Government/ Administration**	e.g., ACT Health, NSW Health, Department of Health and Aged Care.
**Aged Care**	Residential aged care services.
**Educational Facilities/ Research**	e.g., Universities. TAFE
**Other**	Please state:
**Not working at the moment.**	

1.11 Comorbidities: Do you have a chronic condition? This could include, for example, obesity, diabetes, lung disease, auto-immune diseases or another long-term condition.

ANS: Yes, No, Not sure.

1.12 Please indicate if you have any of the following:

Auto-immune Condition/s	Undergoing immunosuppressive medical treatment
Allergy to vaccine/s	History of Guillain-Barre Syndrome
Immunocompromised	Other condition/s I believe prevents me from receiving the flu vaccine.
I do not have any of these conditions	

SECTION 2: Influenza Experience

Attitudes: Vaccine Confidence: Likert Scale

1 – Strongly disagree, 2 – disagree, 3 – neither agree or disagree, 4- agree, 5 – Strongly agree

I believe the flu vaccine is safe.
I believe the flu vaccine is effective in providing protection against the flu.
I would recommend the flu vaccine to others.
Although the flu vaccine appears to be safe, there may be problems that we have not yet discovered.
I worry about the unknown effects of vaccines in the future.
The Flu Vaccines make a lot of money for pharmaceutical companies, but do not do much for regular people.
Authorities promote seasonal influenza vaccination for financial gain, not for people’s health.
The flu vaccine will NOT give me the flu.
Being exposed to diseases naturally is safer for the immune system than being exposed through vaccination.
There is time during my workday to get the flu vaccine.
I trust the TGA to ensure vaccines are safe.
I trust in the authorities to provide reliable information about the risks and benefits of the flu vaccine.
I believe that I could answer patient questions about getting the flu vaccine.
I believe I have a good understanding of how the vaccine protects me from the flu.
HCP are less susceptible to the flu than other people.
I believe the flu shot contains live flu viruses that may cause some people to get the flu.

To your knowledge, have you ever had the flu/ Influenza?

Yes, No, Not sure.

Was this confirmed by a test or GP/ Primary healthcare provider?

Yes, no, not sure.

Perceived Risk: How concerned are you about getting the flu? Would you say…

Not at all concerned,	A little concerned,
Moderately concerned,	Very concerned

Vaccination Behaviour

Vaccination Practice: Have you received the flu vaccine this year? Would you say…

No, not this year, not ever.	Yes, this year, but never before.
No, not this year, but some years.	Yes, this year and some years.
No, not this year, but most years.	Yes, this year and most years.
NOT SURE/ Can’t remember	

Vaccination intention: If you haven't already, Will you get the flu vaccine this year?

Yes, no, not sure.

Will you get the flu vaccine next year?

Yes, No, not sure.

IF you chose YES- to the flu vaccine…

If you have had the flu vaccine, why did you get it? Please choose the most relevant responses (Max 3 answers)

1. I personally believe that having the flu vaccine will protect my health.
2. It is very likely that I can infect my patients with the flu if I don't get the flu vaccine.
3. If I get vaccinated against the flu, then I will be more certain that I will not infect family members.
4. As a healthcare professional it is important that I get the flu vaccine
5. I get the flu vaccine only because I am required to do so.
6. The flu could make me severely ill.
7. If I got the flu, I would feel sicker than most people my age.
8. The thought of getting the flu scares me.
9. It is part of my professional role and a professional responsibility to have the seasonal influenza vaccine.
10. I just do it because my director/ manager recommends it.
11. I would feel bad about myself/ guilty if I didn't get the flu vaccine.
12. It is a job requirement that I must have the flu vaccine.
13. I've carefully thought about flu vaccination and believe it's the right thing to do.
14. As a student, it is part of the university requirement for practical placements.
15. Other. ____

Were there reasons that made it difficult to get the flu vaccine this year? Please choose the most relevant responses (Max 3 answers) - Selected Choice

1. Nothing, it's not hard, [IF NOTHING, SKIP REST OF RESPONSES]
2. Flu vaccination is too expensive.
3. Vaccine is not readily available.
4. Making an appointment is hard.
5. The vaccination site is hard to get to.
6. The vaccination site opening times are inconvenient.
7. The waiting time takes too long.
8. It's difficult to leave work duties.
9. Sometimes people are turned away without vaccination.
10. I didn't get the flu vaccine I didn't want it
11. Is there something else? (ANSWER:______________)

If you chose No, I didn't get it…

Why didn't you get the Flu vaccine this year/ previous years? Please choose the most relevant responses (Max 3 answers)

1. Its not important.
2. I'm young and healthy, I don't need it.
3. I do all the right things...I wash my hands and cover my mouth when I cough.
4. I've had the flu before, it's no big deal.
5. The vaccine will make you sick.
6. The flu is not that serious.
7. The vaccine is more dangerous than the virus.
8. I've never had the flu, so I don't need the flu vaccine.
9. I don't like needles.
10. The flu vaccine doesn't work.
11. It's too late: the flu is already here.
12. Is there something else? (ANSWER:______________)

If you didn't get the Flu vaccine this year/ previous years, were there other reasons that made it hard for you to get the seasonal flu vaccine? Please choose the most relevant responses (Max 3 answers)

- Nothing, it's not hard, [IF NOTHING, SKIP REST OF RESPONSES]
- Flu vaccination is too expensive.
- Vaccine is not readily available.
- Making an appointment is hard.
- The vaccination site is hard to get to.
- The opening times are inconvenient.
- The waiting time takes too long.
- I am unable to leave work duties.
- I don't know where to go to get the flu shot.
- Sometimes people are turned away without vaccination.
- Is there something else? (ANSWER:______________)

Access and Convenience

Do you know where to go to get the Seasonal influenza vaccine? NO/ YES.

Is the seasonal influenza vaccine available for you at your place of work? NO, YES, NOT CURRENTLY WORKING, Not sure/ don't know

How easy is it to get the seasonal influenza vaccine for yourself, if you wanted to?

1 - Not at all easy, 2 - A little easy, 3 – neutral, so-so, 4 -Moderately easy, or 5 - Very easy? Not applicable- I don't want it, I don't know.

Do you get the Flu vaccine for free? YES, NO, Not applicable.

How does your workplace inform you of seasonal influenza vaccination/ vaccine availability? (Check all that apply):

1 Organisational email reminders.		2 Telephone calls	
3 Posters or Brochures		4 Vaccine reminders by text	
5 Manager/ Supervisor reminds me		6 Trolly on the ward/ vaccination hub appears	
7 Nothing, no reminders.		8 Other__________________________	
9 I don't know			

How does your workplace distribute the seasonal influenza vaccine to staff?

1 Organisational email where a link provides a vaccination appointment of yr choice.		2 You must call to make a booking with your OH&S clinic.	
3 Vaccination trolly comes around to the wards, offices.		4 Vaccination hub (drop-in) in central location at workplace	
5 My workplace doesn't provide the vaccine; I have to make our own arrangements.		6 My workplace doesn't provide the vaccine, but I don't want it anyway.	
7 Its available at my work.			
8 Just drop into the OH&S clinic/ vaccine clinic.		9 Vaccination Day at my workplace!	
10 Other, please comment		11 I don't know	

SECTION 3: COVID-19 Experience

To your knowledge, have you ever had COVID-19? Yes, No, Not sure.

Was this confirmed by a test? Yes, no, not sure.

How concerned are you about getting COVID-19?

Not at all concerned,	A little concerned,
Moderately concerned,	Very concerned.

Vaccination Practice: Have you received the Covid- 19 vaccine/s?

No, not ever.	Yes, I have had one of the vaccines.
No, but I plan to.	Yes, I have had a number of vaccines.
	Yes, I have had all the vaccines that I know of/ available to me. (original and boosters)

Will you continue to get COVID-19 vaccines in the future? Yes, No, not sure.

IF you chose YES- to COVID-19 vaccines…

If you have had the COVID-19 vaccine/s, why did you get it? Please choose the most relevant responses (Max 3 answers)

- I personally believe that having the COVID-19 vaccine/s will protect my health.
- It is very likely that I can infect my patients with COVID-19 if I don't get the vaccine.
- If I get vaccinated against COVID-19, then I will be more certain that I will not infect family members.
- As a HCP it is important that I get the COVID-19 vaccine/s.
- I get the COVID-19 vaccine/s only because I am required to do so (Mandated).
- COVID-19 could make me severely ill.
- If I got COVID-19, I would feel sicker than most people my age.
- The thought of getting COVID-19 scares me.
- It is part of my professional role and a professional responsibility to have the COVID-19 vaccine/s.
- I just do it because my director/ manager recommends it.
- I would feel bad about myself/ guilty if I didn't get the COVID-19 vaccine/s.
- I've carefully thought about COVID-19 vaccination and believe it's the right thing to do.
- I want to travel again so its important I have the COVID-19 vaccine.
- Other. ________________________

Were there any other challenges that made it hard for you to get the COVID-19 vaccine?

- Nothing, it's not hard. [IF NOTHING, SKIP REST OF RESPONSES]
- Vaccine is not easily available.
- I don't know how many vaccines (boosters) I'm supposed to have had.
- Making an appointment is hard.
- The vaccination site is hard to get to.
- The opening times are inconvenient.
- The waiting time takes too long.
- I am unable to leave work duties.
- I don't know where to get the COVID-19 vaccine.
- Sometimes people are turned away without vaccination.
- Is there something else? (ANSWER:______________)

If you chose No, I didn't get it…

Why didn't you get the COVID-19 vaccine in the past?

- Its not important.
- I'm young and healthy, don't need it.
- I do all the right things...I wash my hands, wear a mask and cover my mouth when I cough.
- I've had COVID-19 before, it's no big deal.
- The vaccine will make you sick.
- COVID-19 is not that serious.
- The vaccines are more dangerous than the virus.
- I've never had COVID-19, so I don't need the vaccines.

- I don’t like needles.
- The COVID-19 vaccines don’t work.
- It's too late: COVID-19 is already here.
- Is there something else? (ANSWER:_______________)

If you didn’t get COVID-19 vaccine/s, were there other reasons that made it hard for you to get it?

- Nothing, it’s not hard. [IF NOTHING, SKIP REST OF RESPONSES]
- Vaccine is not available.
- Making an appointment is hard.
- The vaccination site is hard to get to.
- The opening times are inconvenient.
- The waiting time takes too long.
- I am unable to leave work duties.
- I don’t know where to get the COVID-19 vaccine.
- Sometimes people are turned away without vaccination.
- Is there something else? (ANSWER:_______________)
